# Effects of exercise programs on cardiopulmonary function and signs and symptoms in patients with post-COVID-19 condition: a systematic review and meta-analysis

**DOI:** 10.3389/fmed.2026.1772741

**Published:** 2026-03-17

**Authors:** Lalita Khuna, Ruschada Sriarmad, Marco Y. C. Pang, Khomkrip Longlalerng

**Affiliations:** 1 Department of Physical Therapy, Faculty of Medicine, Prince of Songkla University, Songkhla, Thailand; 2 Department of Physical Therapy, School of Allied Health Sciences, Walailak University, Nakhon Si Thammarat, Thailand; 3 Movement and Exercise Research Center-Walailak University, Nakhon Si Thammarat, Thailand; 4 Department of Rehabilitation Sciences, The Hong Kong Polytechnic University, Kowloon, Hong Kong SAR, China

**Keywords:** cardiopulmonary function, post-COVID-19, quality of life, rehabilitation, SARS-CoV-2

## Abstract

**Background:**

Exercise is increasingly recognized as an effective adjuvant therapy for individuals with post-COVID-19 condition. However, exercise interventions vary widely in intensity, frequency, setting, and duration. To date, no systematic review and meta-analysis has evaluated programs lasting at least 6 weeks in this population. This study aimed to assess the effects of exercise on cardiopulmonary function and clinical symptoms in patients with post-COVID-19 condition.

**Methods:**

We systematically searched for studies involving patients with post-COVID-19 condition in the Embase, MEDLINE/PubMed, and Scopus databases. The databases were searched using keywords including COVID-19 OR coronavirus OR SARS-CoV-2, AND exercise OR physical exercise OR rehabilitation program, AND pulmonary function OR lung function OR signs and symptoms, AND randomiz* contro* trial OR clinical trial OR RCT on July 2024. The risk of bias of individual trials and the certainty of the body of evidence were evaluated using the Physiotherapy Evidence Database scale and the Grading of Recommendations, Assessment, Development, and Evaluation approach, respectively. The PRISMA (Preferred Reporting Items for Systematic Reviews and Meta-Analyses) statement was used to describe the study selection process. The mean (± standard deviation) for continuous data and the frequency (*n*) and percentage (%) for dichotomous data were estimated, and the effects across trials were combined using a meta-analysis with random-effects models.

**Results:**

We included 10 randomized controlled trials comprising 602 participants. The age of participants ranged from 18 to 70 years. The average exercise duration across the 10 studies was 8.6 weeks (ranging from 6 to 16 weeks). Most exercise programs included aerobic exercise, resistance exercise, breathing exercise, thoracic mobility exercise, chest expansion exercise, and respiratory muscle training. The exercise programs included home-based or telehealth-based programs, center-based programs, and combined center- and home-based programs. Compared with control groups (e.g., usual care, exercise advice, or no structured exercise), exercise interventions significantly improved exercise capacity (6-min walk distance), pulmonary function (forced vital capacity and forced expiratory volume in 1 s), dyspnea (the modified Medical Research Council scale), physical pain, and health-related quality of life domains. The overall certainty of evidence for all outcome measures ranged from moderate to high.

**Conclusion:**

Exercise programs of at least 6 weeks are associated with improved cardiopulmonary function, reduced dyspnea and pain, and enhanced physical and health-related outcomes in patients with post-COVID-19 condition.

**Systematic review registration:**

https://www.crd.york.ac.uk/PROSPERO/view/CRD42024538786.

## 1 Introduction

Coronavirus disease 2019 (COVID-19) is a novel, severe, and contagious viral disease that has been widespread globally since 2019 (1). It is caused by the severe acute respiratory syndrome coronavirus 2 (SARS-CoV-2) (1). As of June 2024, COVID-19 has infected 775,553,735 individuals and caused 7,050,356 deaths worldwide (2). Several body systems are affected by post-COVID-19 condition, including the cardiopulmonary system (e.g., impaired diffusion capacity, interstitial thickening, and lung fibrosis), the gastrointestinal system, the musculoskeletal system, the neurologic system, and psychological manifestations, and others (e.g., dermatologic, ear, nose, and throat symptoms) (3, 4). The incidence of metabolic, cardiovascular, neurologic, and hematologic disorders was also higher following COVID-19 infection than in individuals with no history of COVID-19 infection (5). The most common post-COVID-19 signs and symptoms are fatigue, headache, attention disorder, hair loss, and dyspnea (6). These symptoms can persist for up to 6 months or 1 year after COVID-19 infection (3). Pulmonary rehabilitation is an alternative therapeutic strategy to improve outcomes in patients with acute and post-acute COVID-19 (7). Currently, there is a growing body of evidence on exercise interventions for individuals with post-COVID-19 condition. A wide range of exercise programs has been investigated, including conventional interventions such as aerobic exercise with or without resistance training (8, 9), as well as alternative modalities such as yoga (10), Pilates (11), and Tai Chi (12). Most of these studies have reported improvements in exercise capacity, pulmonary function, and overall quality of life (11, 13–15).

However, considerable variation existed among previous studies in terms of study design, methodology, and participant characteristics, including differences in age, comorbidities, and COVID-19 severity. Additional heterogeneity arose from variations in exercise settings (e.g., home-based, hospital-based, center-based, or telerehabilitation) and in the type, intensity, and duration of the exercise programs (5, 16, 17). Given that the cardiopulmonary system is among the most frequently affected systems in post-COVID-19 condition and typically requires a minimum of 6 weeks of rehabilitation to elicit meaningful physiological adaptations (18, 19), the substantial variability in program duration reported in previous meta-analyses—from as short as 1 week to as long as 7 months—may limit the interpretability of their findings (7, 20). Moreover, those reviews often included a small number of randomized controlled trials and combined inpatient and outpatient populations, further contributing to heterogeneity (7). These variations likely increased between-study heterogeneity, and exercise programs shorter than 6 weeks may have been insufficient to induce the expected physiological responses. Therefore, this study aimed to update and refine the evidence by evaluating the effects of exercise programs lasting at least 6 weeks on cardiopulmonary function and related symptoms among individuals with post-COVID-19 condition.

## 2 Methods

This systematic review and meta-analysis was conducted following the PRISMA (Preferred Reporting Items for Systematic Reviews and Meta-Analyses) guidelines and was registered in the International Prospective Register of Systematic Reviews (PROSPERO) with the ID number CRD42024538786.

### 2.1 Search strategy

All published studies on exercise programs for patients with post-COVID-19 condition from 2019 to July 2024 were identified in three databases, including Embase, MEDLINE/PubMed, and Scopus, with restriction to English-language publications. The key search terms, based on the PICOS framework, included the following components: P (population): “COVID-19” OR “coronavirus” OR “SARS-CoV-2”; I (intervention): “exercise” OR “physical exercise” OR “rehabilitation program”; O (outcomes): “pulmonary function” OR “lung function” OR “signs and symptoms”; and S (study design): “randomiz* contro* trial” OR “clinical trial” OR “RCT.” A tailored search strategy was applied to each database. The full search strategies for all databases are provided in Supplementary Table 1.

### 2.2 Study selection criteria and processes

Studies were included if they met the following criteria: (1) included adult patients (aged >18 years) of any gender with post-COVID-19 condition; (2) were randomized controlled trials (RCTs); (3) implemented an exercise program, pulmonary rehabilitation, or telerehabilitation lasting at least 6 weeks—the minimum duration required to observe adaptations in cardiorespiratory function in pulmonary rehabilitation (18). The exercise program included aerobic exercise, resistance exercise, and/or breathing exercise, or alternative exercise programs (e.g., Pilates and Tai Chi). The programs could be performed using home-based, center-based, hospital-based, or telemedicine platforms; and (4) reported at least one of the following outcome measures: exercise capacity (e.g., 6-min walk distance (6MWD)); pulmonary function (e.g., forced vital capacity (FVC), forced expiratory volume in 1 s (FEV_1_), and FEV_1_/FVC); signs and symptoms, or quality of life (QoL) (e.g., dyspnea scale (modified Medical Research Council (mMRC)), physical pain, physical health, mental health, and emotional role using the Short Form Health Survey-12 or 36 (SF-12, SF-36)).

The exclusion criteria were applied at the study (trial) level. Studies were excluded if they: (1) included patients during hospitalization or during the acute phase of COVID-19 infection; (2) included patients with other comorbidities (e.g., respiratory disease, musculoskeletal disease, and neurologic disease) that may have affected lung function, or included patients who received other treatments (e.g., drugs, nutrition, psychotherapy, and cognitive behavioral therapy); (3) implemented exercise programs lasting less than 6 weeks; (4) were cohort studies, case–control studies, cross-sectional studies, case reports, literature reviews, or systematic reviews; or (5) did not provide detailed data in the original paper for further analysis.

After the removal of duplicates, titles and abstracts of identified studies were independently reviewed by two investigators with experience in systematic reviews. Studies that did not meet the eligibility criteria were excluded. Then, the full texts of the remaining articles were independently reviewed by two reviewers. If there was disagreement, the articles were reviewed by a third reviewer. Inter-rater agreement was assessed using Cohen’s kappa coefficient. The level of agreement was interpreted according to the Landis and Koch classification (21).

### 2.3 Data extraction

Data were extracted independently by two researchers (KL and LK) using a standardized form. The extracted information included the author name, year of publication, country, study design, sample size, participants’ demographic data, interventions of the experimental and control groups, frequency and duration of the interventions, and outcome measurements. In case of any disagreements, there was discussion with a senior supervisor until we reached consensus.

### 2.4 Assessment of risk of bias

The studies were evaluated independently by two reviewers (KL and LK), who used the PEDro scale to rate the methodological quality of the included trials. The scale consists of 11 criteria, each rated as “yes” or “no.” One of these criteria addresses external validity, while the other 10 contribute one point each, if fulfilled, resulting in a total possible score of 10. Trials scoring less than 4 are classified as “poor,” those scoring between 4 and 5 are labeled “fair,” scores from 6 to 8 are deemed “good,” and scores between 9 and 10 are rated as “excellent” (22). The PEDro score has demonstrated moderate to excellent inter-rater reliability (intraclass correlation coefficient [ICC] = 0.53 to 0.91) for clinical trials of physiotherapy-related interventions (22).

### 2.5 Assessment of the certainty/quality of evidence

The certainty of the evidence across trials was evaluated using the Grading of Recommendations, Assessment, Development, and Evaluation (GRADE) approach. The evidence was rated as high, moderate, low, or very low certainty. The GRADE approach was applied based on risk of bias (PEDro score; downgraded when the average PEDro score was <6), inconsistency (heterogeneity, I^2^ > 50%), indirectness, imprecision (confidence interval width), and publication bias (funnel plot, Egger’s test), with a focus on the 6MWD, FVC, FEV_1_, and mMRC as the key outcomes. Two authors independently assessed the certainty of the evidence (KL and LK), while the third author determined the consensus in case of any disagreement.

### 2.6 Data analysis

Outcome measurement data were classified into three categories, including (1) exercise capacity (6MWD), (2) pulmonary function (FVC, FEV_1_, FEV_1_/ FVC), and (3) signs and symptoms or QoL (e.g., physical health, physical pain, mental health, and emotional role domains). The mMRC dyspnea scale is an ordinal measure ranging from 0 to 4. For the purpose of meta-analysis and to facilitate clinically meaningful interpretation, we dichotomized mMRC scores using a predefined threshold of ≥2 to indicate clinically significant dyspnea, while scores of 0–1 were classified as no or mild dyspnea. All data were analyzed using the STATA program (version 18.0, StataCorp, TX, USA). For continuous data, the mean difference (MD) with corresponding 95% confidence intervals (CIs) was calculated for each outcome using post-intervention means and standard deviations (SDs), allowing comparison between the intervention and control groups when outcomes were measured using the same instrument and scale across studies. In this study, change-from-baseline data were not consistently reported, and the calculation of their SDs required correlation coefficients that were unavailable. As baseline characteristics were comparable between groups, the use of post-intervention values was considered appropriate. For dichotomous data, the treatment effect between groups was presented as a risk ratio (RR) with 95% CI. Whether a fixed or a random-effects model should be adopted was determined based on the results of the heterogeneity among trials. An I^2^ statistic was used to assess the statistical heterogeneity of the included studies (<25, 25–75%, >75% representing low, moderate, and high heterogeneity) (23). Subgroup analyses were conducted in cases of moderate to high heterogeneity (I^2^ > 25%) (e.g., duration, age, and setting). For exercise setting, interventions were categorized as home-based (including telerehabilitation or remotely supervised programs conducted at home) and non-home-based (center-based or facility-based programs). This subgroup analysis was performed to explore potential differences in intervention delivery models. If the I^2^ value decreased by more than 50%, it suggested that the variable might have influenced the outcomes. A sensitivity analysis was performed by removing the studies that had the greatest influence on the forest plot. Publication bias across the included studies was evaluated using funnel plots and Egger’s test, but only for 6MWD, FEV_1_, FVC, and mMRC due to the limited number of studies. The significance of the pooled index was set at *p* < 0.05, and all tests were two-sided.

## 3 Results

**Figure 1:**
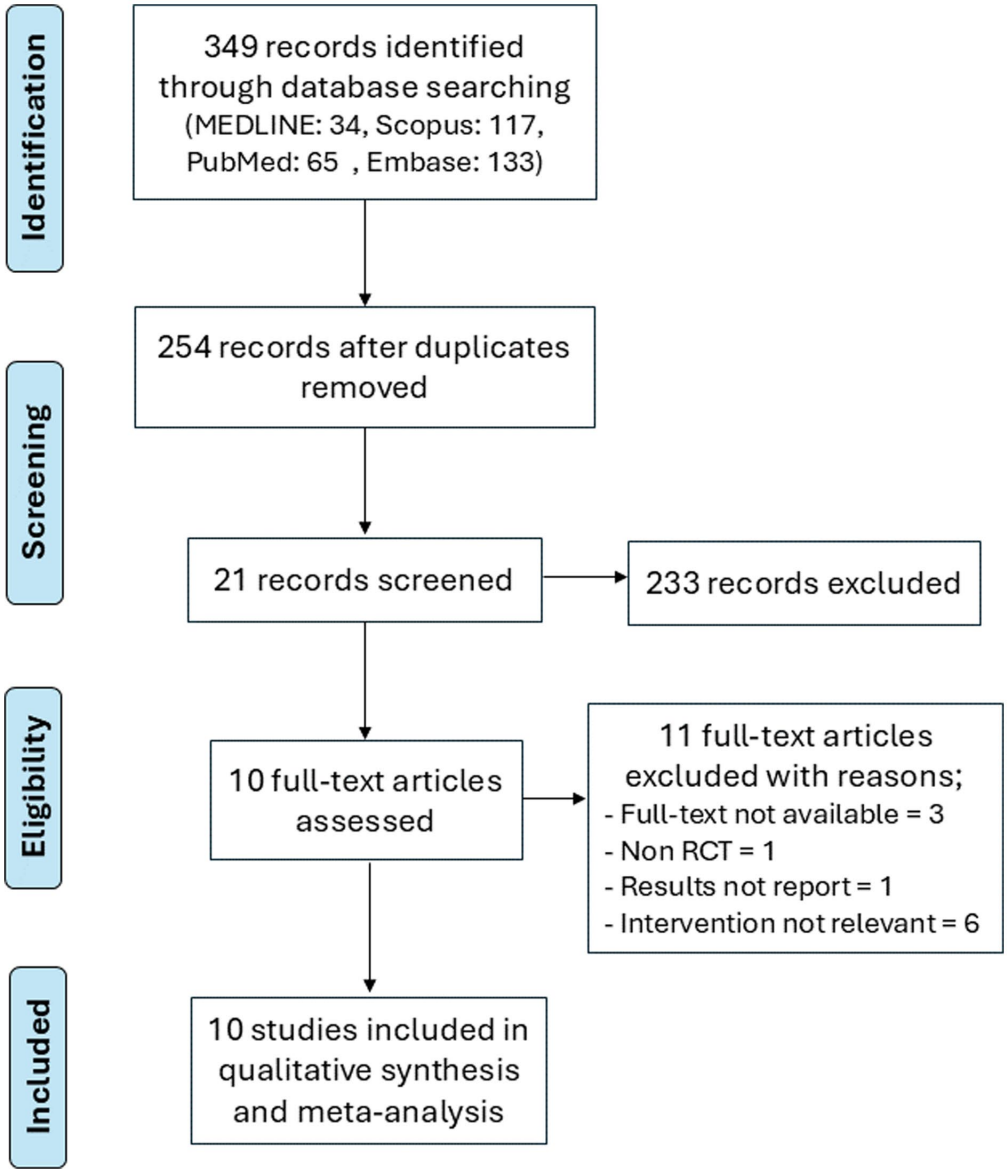
Flow chart of the study selection.

The literature search retrieved 349 studies from three databases. After screening the title and abstracts, 21 studies were chosen for full-text review. The agreement between the two independent reviewers was 89%, with a Cohen’s kappa of 0.496, indicating moderate agreement. Discrepancies were resolved through discussion, and when consensus could not be reached, a third reviewer was consulted for adjudication. Ten trials met the eligibility criteria and were included in the review (13, 14, 24–31). The flow diagram of the study selection process is illustrated in Figure 1.

### 3.1 Risk of bias assessment

Of these studies, three were categorized as excellent quality (13, 26, 27) and seven as good quality (14, 24, 25, 28–31) (Supplementary Table 2).

### 3.2 Characteristics of included studies

The characteristics of the 10 included trials are presented in Table 1. A total of 602 participants were included, comprising 298 men (49.5%) and 304 women (50.5%). The age range of all included participants was 18–70 years, with a mean age of 52 years. The severity of illness among participants ranged from mild to very severe. All participants had been discharged from the hospital.

**Table 1:** Characteristics of included studies.

Author, year, country	Population	Severity	Comorbidities	Sample size (IG/CG)	Intervention setting/mode	Exercise protocol	Duration (weeks)	Control	Outcomes	Results	PEDRro score
Alsharidah et al., 2023 (13), Saudi Arabia	Young female aged 18–30 years	Mild to moderate (OPD cases)	Not reported, most of the patients were young	48 (24/24)	Telerehabilitation pulmonary exercise program	Breathing exercises and chest expansion exercises for 15 min AE for 20–30 min, 60–80% MHR RT for 30 min, 10RM Three sessions/week Total time 60–80 min/session	6	Suggest performing regular everyday routines, prescribed educational presentations	6MWD, FEV_1_, FVC, HRQoL (SF-36)	6MWD and HRQoL were significantly improved in the intervention group compared with the control group (*p* = 0.001)	10
da Silva et al., 2025 (24), Brazil	After hospital discharge, hemodynamically stable; no tracheostomy; not bedridden; no decompensated chronic diseases or neuromuscular and orthopedic diseases	n/a (mostly severe to very severe because admitted in ICU)	Mostly CVD (65%), DM, pulmonary disease, and obesity (60%)	57 (28/29)	Telerehabilitation supervised by a physical therapist (videoconference)	Warm-up with breathing and thoracic mobility exercises AE for 30 min, RPE scale at 3–4 of 10, and 70–80% MHR RT at 50–100% of 10RM for upper limbs, and 100–200% of the 30CST for lower limbs ST Relaxation and breathing exercises Three sessions/week Total duration 30–50 min/session	8	General care and self-monitoring of vital signs (videoconference)	6MST, 2MSWT, 30CST, SF-36 (physical functioning domain)	6MST (*p* < 0.05), 2MSWT (*p* = 0.001), and HRQoL—physical functioning (*p* < 0.001) were significantly improved in the intervention group compared with the control group	6
Elyazed et al., 2024 (14), Egypt	Aged 40–70 years, after hospital discharge or home treatment, no ICU admission	n/a (no severe)	All patients had obesity but no chronic disease	60 (30/30)	Home exercises with telemonitoring and face-to-face supervision	Regular walking for 30–60 min, 5 days a week, normal pace RMT for 10–15 min, two times per day, minimal load of 1–2 kg placed on the abdomen RT with low weight 1–3 kg, three sets with 10 reps, two times a day, 5–7 days a week, low weight of 1–3 kg	12	No exercise program with usual medical care	6MWD, PFI, CFS, mMRC, SF-36	CFS, 6MWD, PFI, SF-36, and mMRC were significantly improved in the intervention group compared with the control group (*p* < 0.001)	7
Fares et al., 2023 (25), Egypt	Aged 50–70 years	Post-severe	DM, HT, and obesity	100 (50/50)	Pulmonary rehabilitation program (outpatient clinic combined with home-based training)	Breathing exercises (pursed-lip breathing, diaphragmatic breathing, and incentive spirometry) Circuit exercise program (stretching and body-weight exercises) Walking exercise, 1–30 min/time, 1–5 sessions/day (gradually progress using time/session of each day) Six sessions/week (three outpatient clinic sessions and three home sessions)	6	Usual medical care	mMRC, FVC, 6MWD	mMRC, FVC, and 6MWD were significantly improved in the intervention group compared with the control group (*p* < 0.001)	7
Jimeno-Almazán et al., 2023 (26), Spain	Non-hospitalized patients with post-COVID-19 condition, aged >18 years, with a chronic symptomatic phase lasting >12 week from symptom onset	n/a (no severe)	Mood disorders (*n* = 34), but many chronic diseases were excluded	43 (23/20) Note; Total *n* = 80 from four groups, *n* = 23 received concurrent exercise and respiratory exercise	A tailored multicomponent exercise program adapted from the ACSM guideline with inspiratory muscle training (center-based)	Concurrent training with RMT for 3 days/week (2 days of RT, 50%1RM, and 1 day of light intensity continuous training, 30–60 min), moderate-intensity variable training (MIVT): 4–6×3–5 min at 70–80% of HRR Light intensity continuous training (LICT: 30–60 min, 65–70% HRR) 11–16 of Borg score IMT (1 set of 30 repetitions, two times/day, 62.5 ± 4.6% of maximal inspiratory pressure (MIP), 12–15 of Borg scale)	8	Following the WHO guidelines for post-COVID-19-related illness rehabilitation	VO_2_max, muscle strength, SF-12, GAD-7, PHQ-9, mMRC, FSS, CFS	SF-12, mMRC, FSS, CFS, PHQ9, and GAD7 were significantly improved in the intervention group compared with baseline. However, no significant differences were found in any other variable between groups	9
Kaddoussi et al. (27), 2024, Tunisia	Aged > 18 years, mMRC≥2 for more than 3 months	Various severities ranging from mild to very severe (post-ICU)	DM, HT, dyslipidemia, and thyroid disorders	30 (20/10)	Exercise training (outpatient department)	5-min warm-up AE (walking) for 10–35 min, light intensity (increase time by 5 min/week), HR target = HREnd ± 5 bpm (from 6MWT) RT for 15–20 min, 10 reps, three sets/time (body weight and dumbbell) Respiratory exercise for 15 min Three sessions/week, 60–90 min in each session	6	Usual level of sedentary physical activities	mMRC, FEV_1_, FVC, FEV_1_/FVC, 6MWD, SpO_2_, heart rate	mMRC (*p* = 0.001) and 6MWD (*p* = 0.023) were significantly improved in the intervention group compared with the control group	9
Li et al., 2022 (28), China	Aged 18–75 years, post-discharge COVID-19 patients with an mMRC score of 2–3	Both severe and non-severe cases	61% had at least one comorbidity (e.g., HD, HT, DM, obesity, TB, others)	119 (59/60)	Home exercise program via a smartphone application (RehabApp)	Breathing control and thoracic expansion exercises, 2–3 sets, 12 reps, 10–15 min AE at 30–60%HRR, RPE 11–14, 20–30 min RT (body weight), 12 reps, 2–3 sets, 9–15 min Three to four sessions/week	6	Received short educational instructions	6MWD, lower limb muscle strength, FEV_1_, FVC, FEV_1_/FVC, PEF, MVV, SF-12, mMRC	6MWD, muscle strength, MVV, SF-12, and mMRC were significantly improved in the intervention group compared with the control group (*p* < 0.01)	8
Liu et al., 2020 (29), China	Older adults aged > 65 years, 6 months after infection	n/a (tended to be severe because all were post-hospital discharge with a variety of comorbidities)	HT, DM, and osteoporosis	72 (36/36)	Respiratory rehabilitation training (home-based)	RMT (ThresholdPEP), 10 breaths/set, three sets, 60% of maximum expiratory pressure Cough exercise, 10 times, three sets Diaphragmatic training, 30 times, add weight 1–3 kg ST Home exercise: pursed-lip breathing and coughing (30 sets/day) Two sessions/week	6	Not reported	FEV_1_, FVC, FEV_1_/FVC, DLCO, 6MWD, SF-36, FIM, SAS, SDS	FEV1, FVC, FEV1/FVC, DLCO, 6MWD, SF-36, and SDS were significantly improved in the intervention group compared with the control group (*p* < 0.05)	6
Longobardi et al., 2023 (30), Brazil	Aged > 45 years, discharged from the ICU 3–6 months before study; half of the patients required MV	Severe/critical	HT, dyslipidemia, rheumatic disease, DM, CVD, psychological disorders, pulmonary disease, hypothyroidism, others; all patients had obesity (100%)	41 (21/20)	Semi-supervised, individualized, home-based exercise training	AE (walking/jogging 10 min to ≥50 min/day) using Borg 9–17 RT, 8–15 reps, 3–5 sets, intensity progressed every 2 weeks using Borg 9–17 ST for the major muscle groups Three sessions/week (~60–80 min/session)	16	Standard of care	SF-36, FEV_1_, FVC, FEV_1_/FVC, cardiorespiratory fitness, handgrip strength, 30CST, TUGT, FSS, BAI, BDI	Only the 30CST showed no significant difference between the intervention and control groups (*p* = 0.48)	7
Teixeira et al., 2022 (31), Brazil	Aged ≥18 years, after hospital discharge	Both severe and non-severe	HT, DM, CVD, hypothyroidism, respiratory disease, obesity, others	32 (12/20)	Tele-supervised home-based exercise training	5-min warm-up RT (3 days/week on alternate days) one set of 10–15 reps (week 1), two sets of 10–15 reps (weeks 2–3), three sets of 10–15 reps (weeks 4–6), and three sets of 15–20 reps (weeks 7–12), intensity 14–17 of RPE AE (5 days/week) 10–15 min (week 1), 20 min (weeks 3–4), and 30 min (weeks 5–12), intensity 11–13 of RPE 5-min cool-down	12	Not reported	FEV_1_, FVC, FEV_1_/FVC, MIP, MEP, handgrip strength, FTSST, TUGT, 6MWD	FEV1, FVC, and handgrip strength improved significantly (*p* < 0.001). Only the intervention group showed improvements in MIP and MEP compared with baseline. However, no significant differences were found in any other variable between groups	6

ACSM, American College of Sports Medicine; AE, aerobic exercise; BAI, Beck Anxiety Inventory; BDI, Beck Depression Inventory; CFS, Chalder Fatigue Scale; CG, control group; CVD, cardiovascular disease; DLCO, diffusing capacity of the lung for carbon monoxide; DM, diabetes mellitus; FEV1, forced expiratory volume in 1 s; FVC, forced vital capacity; FIM, Functional Independence Measure; FSS, Fatigue Severity Scale; FTSST, five-times sit-to-stand test; GAD-7, General Anxiety Disorder-7; HRQoL, health-related quality of life; HRR, heart rate reserve; HT, hypertension; ICU, intensive care unit; IG, intervention group; MEP, maximal expiratory pressure; MHR, maximum heart rate; MIP, maximal inspiratory pressure; mMRC, Modified Medical Research Council Dyspnea Scale; MVV, maximum voluntary ventilation; MV, mechanical ventilation; n/a, not applicable; PFI, Physical Fitness Index; PHQ-9, Patient Health Questionnaire-9; PEF, peak expiratory flow; reps, repetitions; RM, repetition maximum; RMT, respiratory muscle training; RPE, rating of perceived exertion; RT, resistance exercise; SAS, Self-Rating Depression Scale; SDS, Self-Rating Anxiety Scale; 6MWD, 6-min walk distance; 6MST, 6-min step test; SF-36, Short Form Health Survey-36; ST, stretching exercise; 30CST, 30-s chair stand test; TB, tuberculosis; TUGT, timed up and go test; 2MSWT, 2-min stationary walk test.

### 3.3 Details of interventions

The treatment protocol in all included studies is presented in Table 1. The average exercise duration was 8.6 weeks (ranging from 6 to 16 weeks). Most exercise programs included aerobic exercise (nine trials), resistance exercise (nine trials), respiratory muscle training (three trials), breathing exercise and relaxation techniques, chest expansion exercise, or thoracic mobility exercise (five trials), incentive spirometry (one trial), and stretching exercise. The exercise settings included home-based programs (14, 28, 29, 31) or telerehabilitation programs (13, 24, 31), a combination of home-based and hospital-based programs (25, 30), and center-based or hospital-based programs (26, 27). Only four studies explicitly reported that no adverse events occurred during the intervention period. The remaining studies did not provide information regarding safety outcomes. Three studies reported exercise adherence in the intervention group, which ranged from 71 to 94%. For the control group, five studies recommended regular exercise or provided exercise-related education. Three studies instructed participants to receive usual care or not to exercise, and two studies did not report any exercise recommendations.

### 3.4 Outcomes

The effects of the exercise program on the outcome measures are shown in Table 1.

#### 3.4.1 Exercise capacity

**Figure 2:**
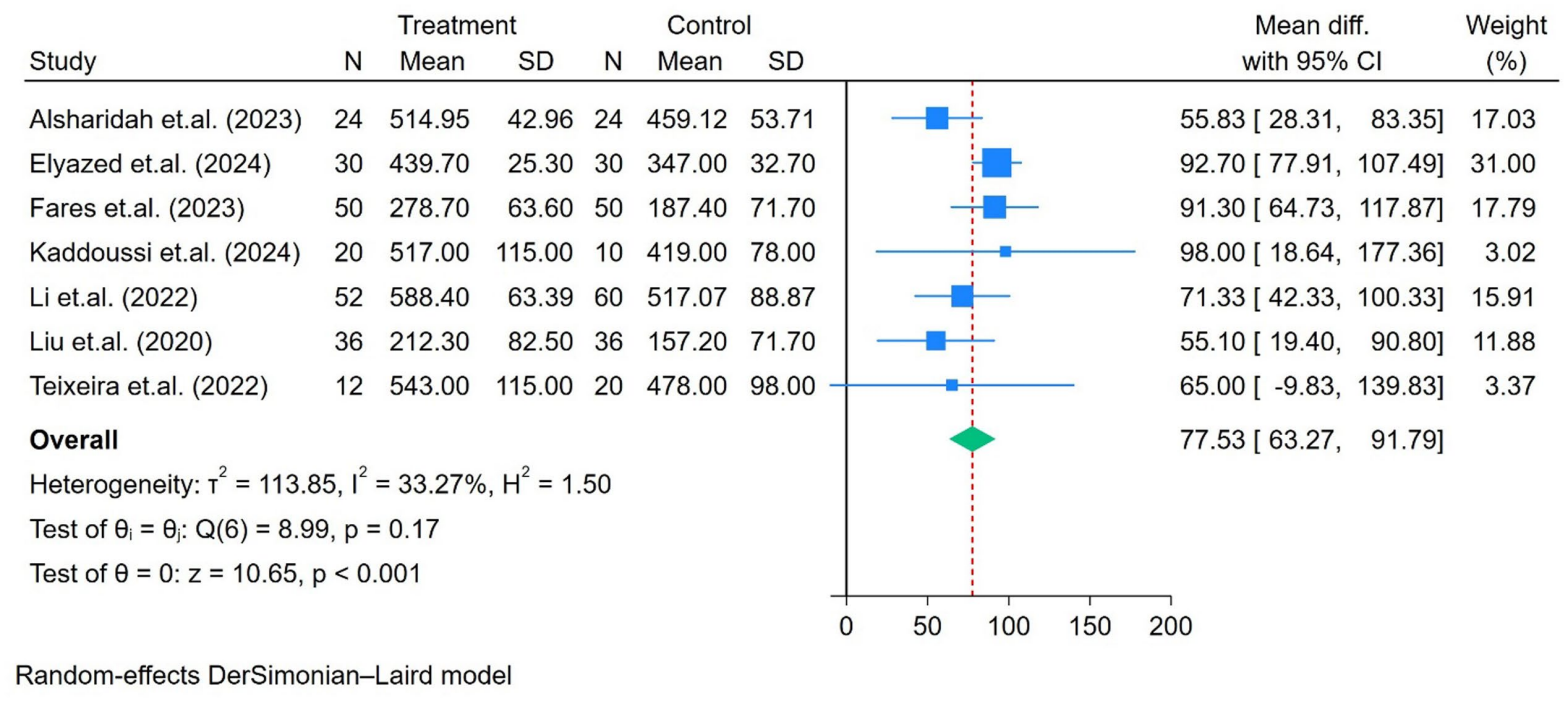
Forest plot of the seven studies reporting 6MWD (m) comparing exercise and control groups.

The exercise capacity was reported in seven studies, all of which were determined using the 6MWD. As shown in Figure 2, moderate heterogeneity was observed among the seven studies (*p* = 0.17, I^2^ = 33%), and a random-effects DerSimonian–Laird model was applied. An improvement in 6MWD was observed after the intervention (mean difference = 77.5 m, *p* < 0.01; Figure 2). After conducting a subgroup analysis based on the duration of the intervention (6 weeks *vs*. > 6 weeks), the heterogeneity decreased by more than 50%, as shown in Supplementary Figure 1. The 6MWD improved by 71.1 m in programs lasting 6 weeks and by 91.7 m in programs lasting longer than 6 weeks. The *p*-value between the groups showed an almost significant difference (*p* = 0.06). In addition, a subgroup analysis based on the exercise setting (home-based *vs*. non-home-based) demonstrated a significant difference in 6MWD improvement, with non-home-based programs improving by 92.4 m compared with 61.4 m in home-based programs (*p* < 0.001), as shown in Supplementary Figure 2. A sensitivity analysis was performed by excluding the study with the youngest patients (13), revealing a greater improvement in 6MWD (85 m) (I^2^ = 3.43%, *p* < 0.01; Supplementary Figure 3).

#### 3.4.2 Pulmonary function test

**Figure 3:**
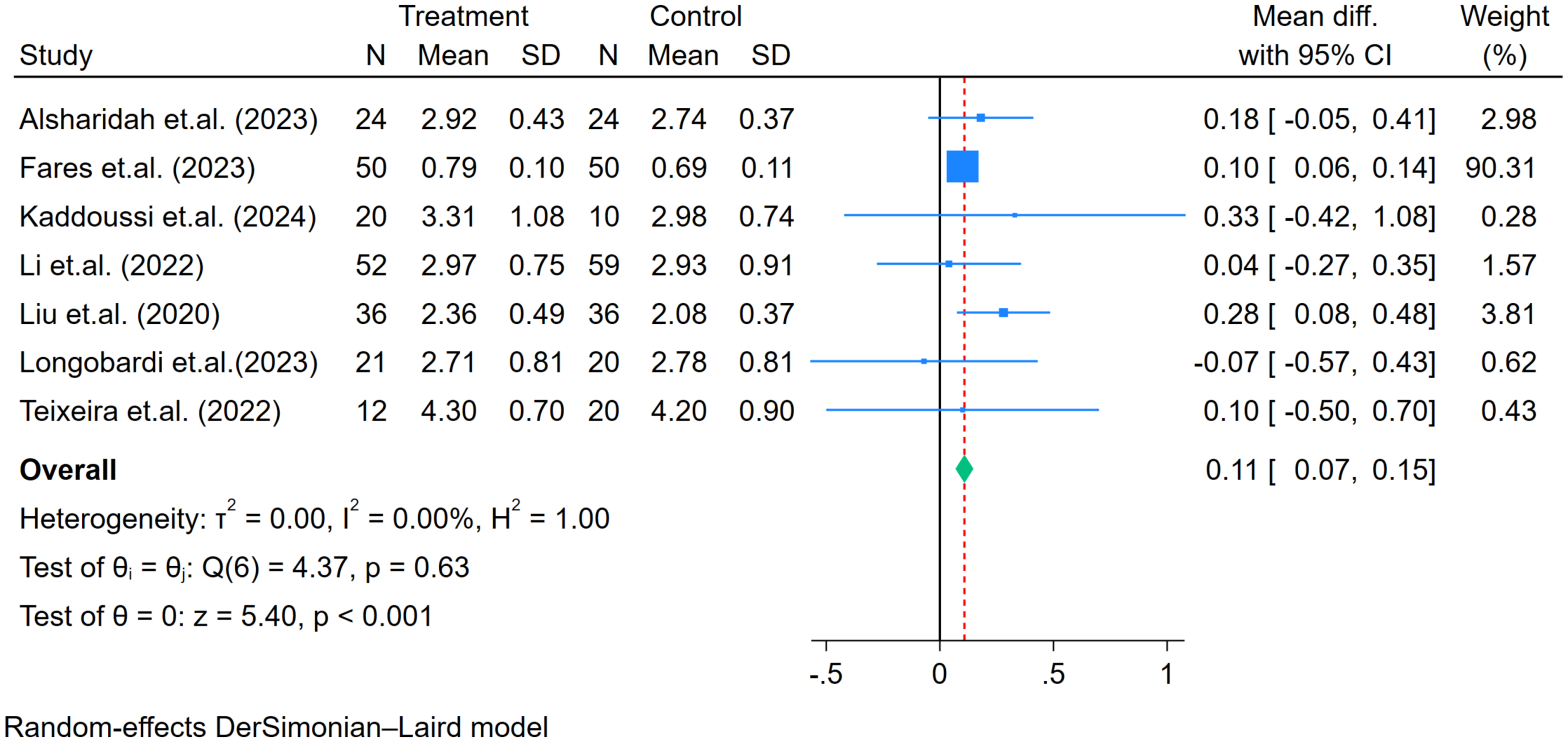
Forest plot of the seven studies reporting FVC (L) comparing exercise and control groups.

**Figure 4:**
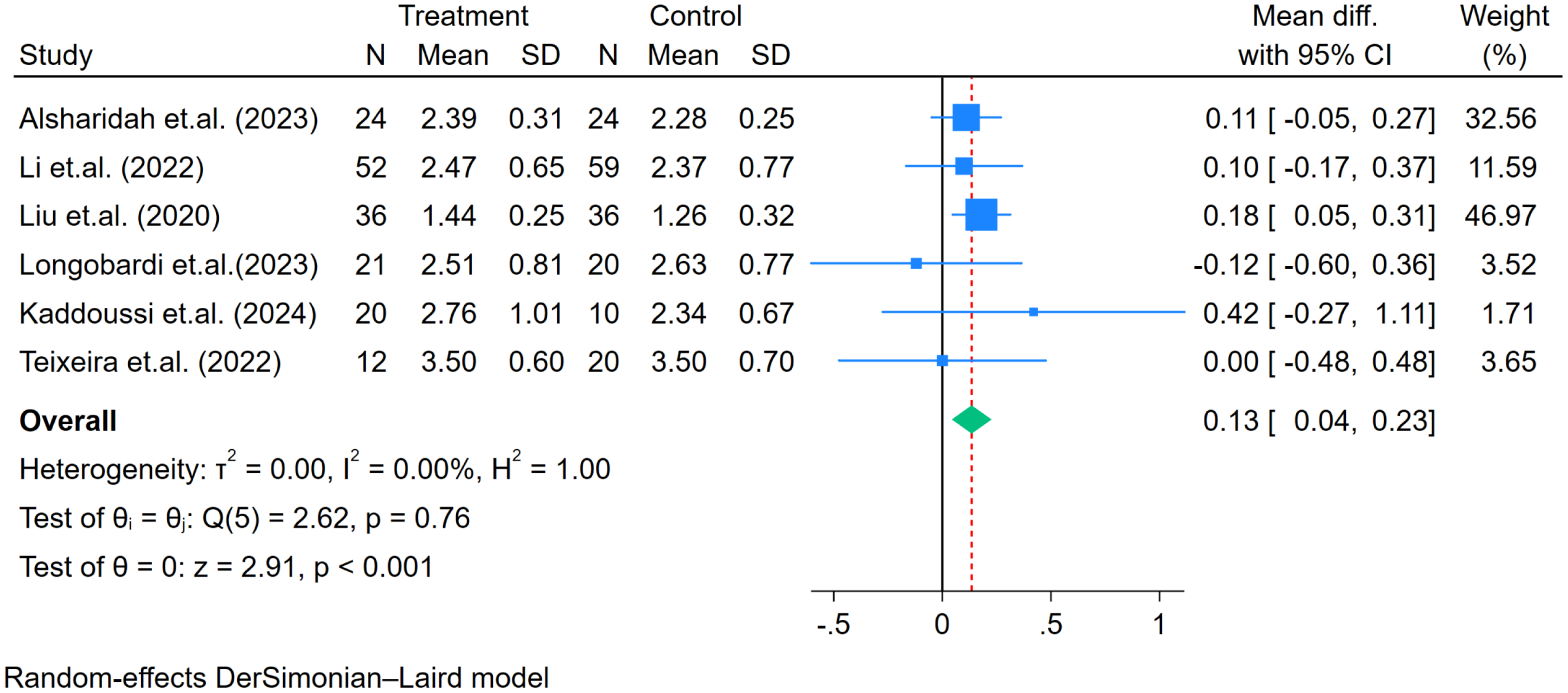
Forest plot of the six studies reporting FEV_1_ (L) comparing exercise and control groups.

**Figure 5:**
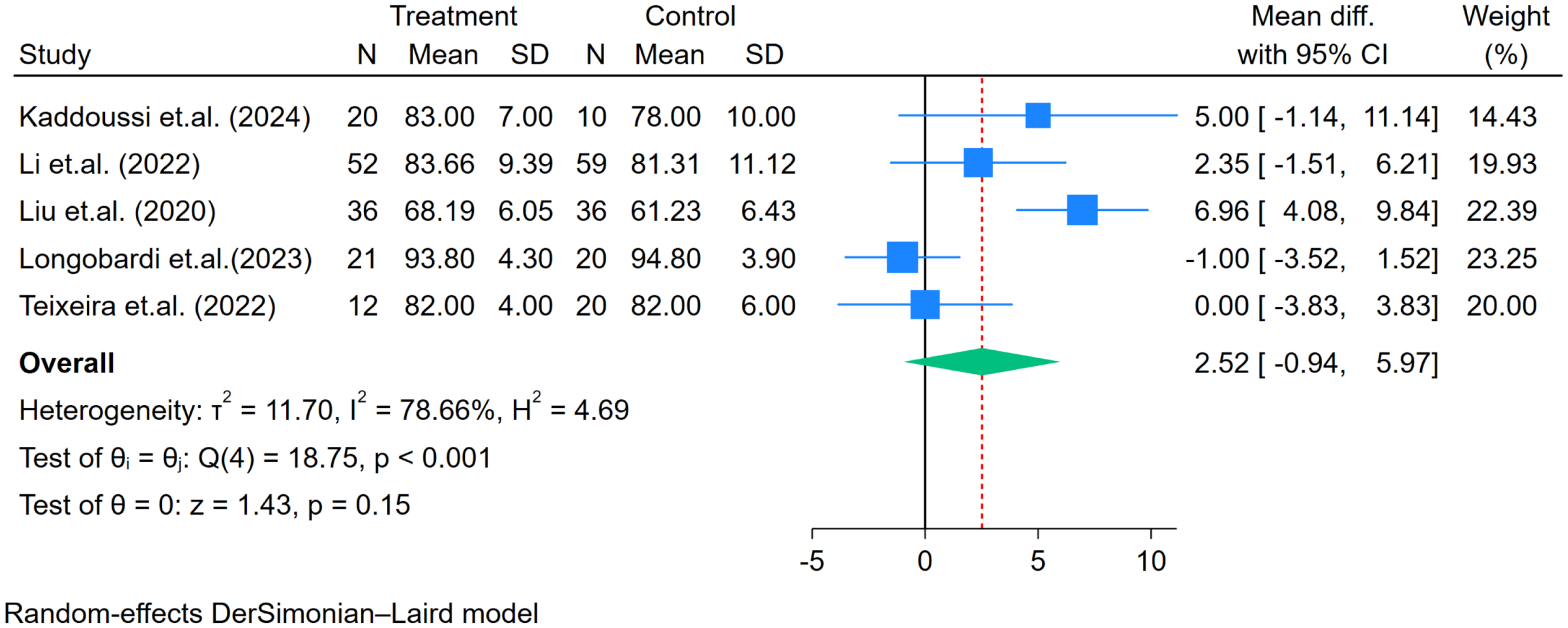
Forest plot of the five studies reporting FEV_1_/FVC (%) comparing exercise and control groups.

Seven, six, and five studies assessed the FVC, FEV_1_, and FEV_1_/FVC, respectively (Figures 3–5). No heterogeneity for FVC or FEV_1_ was reported in the selected studies (I^2^ for FVC and FEV_1_ was 0%). The mean difference of FVC and FEV_1_ showed significant improvement after the exercise intervention (FVC = 0.11 L, *p* < 0.01; FEV_1_ = 0.13 L, *p* < 0.01; Figures 3, 4). The FEV_1_/FVC showed no significant difference between the exercise and control groups (FEV_1_/FVC = 2.52%, *p* = 0.15, with high heterogeneity (I^2^ = 79%); Figure 5).

#### 3.4.3 Signs and symptoms or quality of life [dyspnea scale (mMRC), physical pain, physical health, mental health, and emotional role domains] (SF-36)

**Figure 6:**
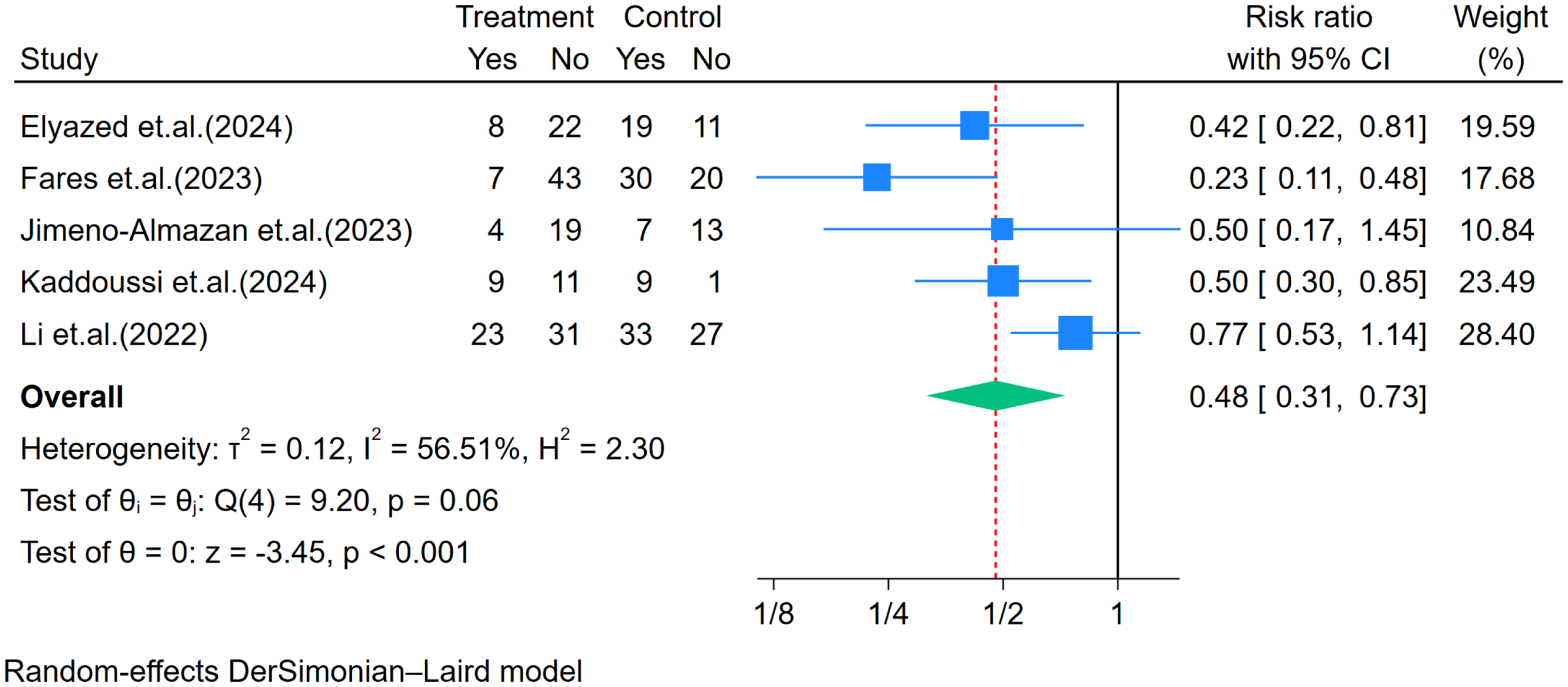
Forest plot of the five studies reporting dyspnea (mMRC) comparing exercise and control groups.

Five studies assessed the mMRC (Figure 6). Moderate heterogeneity was observed among the five studies (*p* = 0.06, I^2^ = 56.51%). The dyspnea level significantly improved after the exercise intervention (RR = 0.48, *p* < 0.01; Figure 6). The difference in exercise duration among the studies was further analyzed through subgroup analysis, which showed no significant difference in mMRC between the groups (*p* = 0.85). The sensitivity analysis indicated that one study influenced the overall effect size (28). However, the findings remained significant, showing an improvement in mMRC (I^2^ = 2%) with an effect size of 0.40 (95% CI: 0.29–0.57), *p* < 0.01.

**Figure 7:**
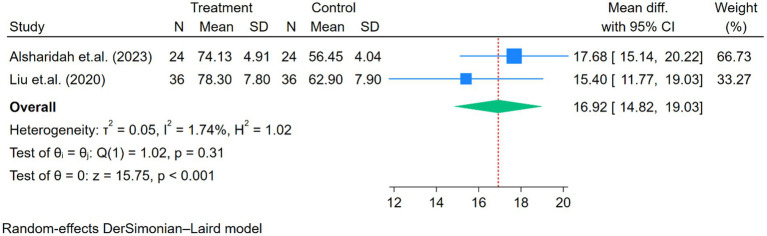
Forest plot of the two studies reporting physical pain comparing exercise and control groups.

**Figure 8:**
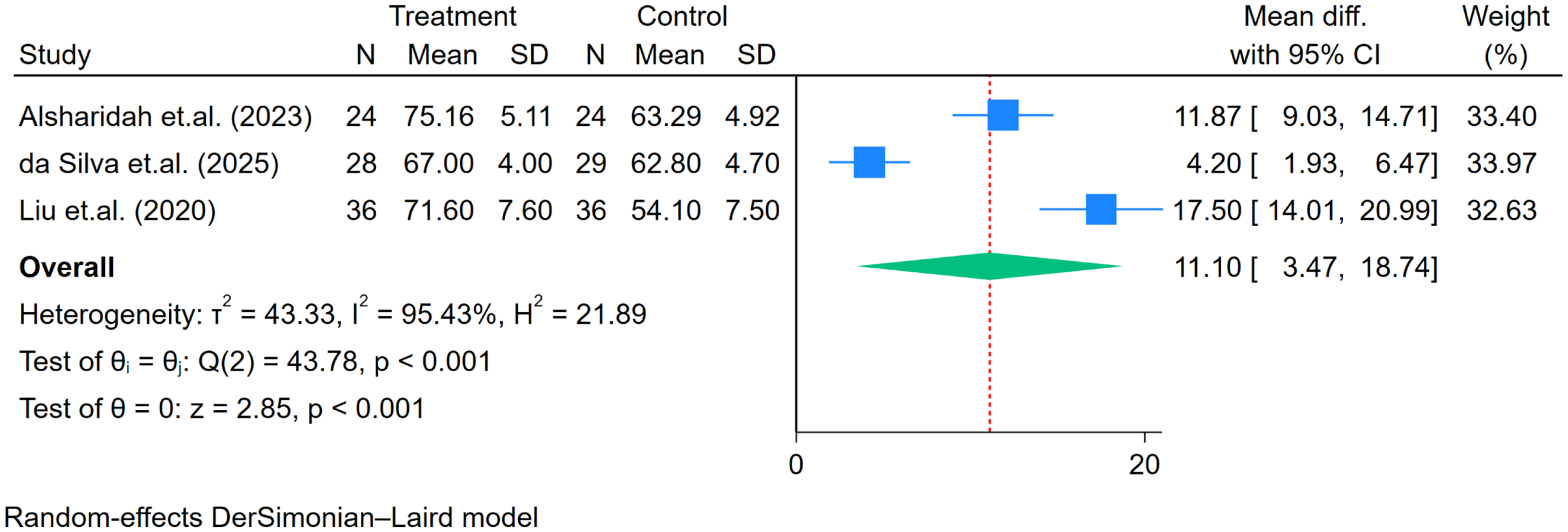
Forest plot of the three studies reporting physical health comparing exercise and control groups.

**Figure 9:**
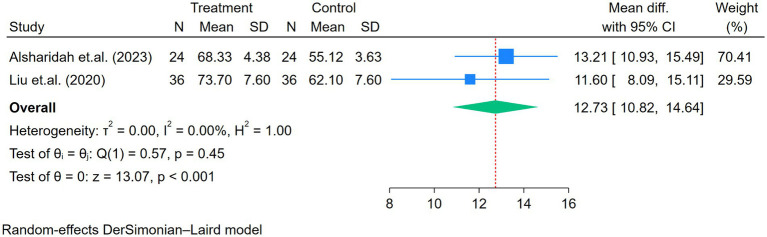
Forest plot of the two studies reporting mental health comparing exercise and control groups.

**Figure 10:**
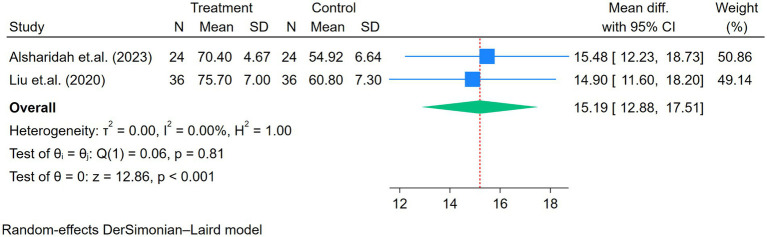
Forest plot of the two studies reporting emotional role comparing exercise and control groups.

Other signs and symptoms, including physical pain, physical health, mental health, and emotional role, showed significant improvement after the exercise program (all *p* < 0.01; Figures 7–10).

### 3.5 Publication bias

The funnel plot for the 6MWD, FVC, FEV_1_, and mMRC showed no indications of publication bias [Z = −0.66 (continuity corrected), Pr > |z| = 0.51 for 6MWD; Z = 0.63 (continuity corrected), Pr > |z| = 0.53 for FVC; Z = −0.57 (continuity corrected), Pr > |z| = 0.57) for FEV_1_; Z = −1.66 (continuity corrected), Pr > |z| = 0.09) for mMRC; Supplementary Figures 4–7]. However, these findings should be interpreted with caution, as the number of included studies for each outcome was fewer than 10, limiting the statistical power and reliability of the publication-bias assessment.

### 3.6 Certainty/quality of evidence using the GRADE approach

This meta-analysis included only RCTs, representing a high-quality study design. Study quality was evaluated across five GRADE domains. First, no downgrading was applied for risk of bias because all included trials achieved a PEDro score greater than 6. Second, consistency was judged to be high for the primary outcomes—6MWD, FVC, and FEV₁—which demonstrated low heterogeneity and consistent effects across studies. In contrast, several secondary outcomes, such as symptom-related measures, showed higher heterogeneity and were based on fewer studies, resulting in lower certainty. Third, directness was rated as high because all trials involved individuals with post-COVID-19 condition, used broadly similar exercise interventions (aerobic and/or resistance training), and assessed comparable primary outcomes. Fourth, imprecision led to downgrading for several secondary outcomes: physical pain, physical health, mental health, and emotional role, which were characterized by small sample sizes and wide CIs. Finally, no publication bias was detected for 6MWD, FVC, FEV₁, or mMRC.

Overall, high-certainty evidence from seven trials (454 participants) showed that exercise programs lasting at least 6 weeks led to clinically important improvements in 6MWD (MD 77.5 m, 95% CI 63.3 to 91.8). Similarly, high-certainty evidence from seven trials (443 participants) indicated improvements in FVC (MD 0.11 L, 95% CI 0.07 to 0.15). Six trials (343 participants) also provided high-certainty evidence for increases in FEV₁ (MD 0.13 L, 95% CI 0.04 to 0.23). For dyspnea, moderate-certainty evidence from five trials (357 participants) demonstrated a reduction in mMRC scores (RR 0.48, 95% CI 0.31 to 0.73), suggesting that exercise may meaningfully alleviate breathlessness. Certainty ratings for all outcomes are summarized in Supplementary Table 3.

## 4 Discussion

This systematic review and meta-analysis was the first to evaluate the effects of exercise programs lasting at least 6 weeks on cardiopulmonary function and symptom burden in patients with post-COVID-19 condition. Across 10 RCTs, exercise interventions consistently improved functional exercise capacity (6MWD), pulmonary function parameters (FVC and FEV_1_), and multiple patient-reported signs and symptoms, encompassing dyspnea severity (mMRC scale), physical pain, physical health status, mental health, and emotional wellbeing domains. Together, these findings support the growing recognition that structured exercise is a viable and effective rehabilitation strategy for individuals with post-COVID-19 condition.

A key inclusion criterion in this meta-analysis was that participants engaged in an exercise program for at least 6 weeks, a duration widely recognized in exercise physiology as necessary to elicit measurable aerobic adaptations (32). The pooled analysis demonstrated a clinically meaningful improvement in 6MWD. Importantly, the magnitude of change exceeded the minimal clinically important difference (MCID) reported for chronic respiratory diseases such as COPD (36.3 m) (33) and idiopathic pulmonary fibrosis (22–37 m) (34), underscoring its clinical relevance. Previous studies in individuals recovering from COVID-19 have reported improvements ranging from 44.2 to 76.5 m (7, 20, 35), which align with the present findings. Although the previous systematic reviews and meta-analyses differed in several important aspects—such as the limited number of available studies during the early phase of the pandemic (20), variation in disease severity, differences in patient characteristics (e.g., age and comorbidities), and heterogeneity in exercise program parameters (7, 20, 35)—their findings were broadly comparable with those of this study.

In these previous reviews (7, 20, 35), aerobic exercise was the most commonly prescribed intervention, followed by resistance training and respiratory muscle training, which is consistent with this study. For aerobic exercise, intensity in the previous reviews was prescribed using 50–75% of maximum heart rate (MHR), 30–80% of heart rate reserve (HRR), or rating of perceived exertion (RPE) (modified Borg scale: 6–8/10; Borg scale: 9–17/20), whereas in this study it was prescribed at 60–80% of MHR, 30–70% of HRR, or RPE (modified Borg scale: 3–4/10; Borg scale: 9–17/20). Nevertheless, moderate intensity was most commonly prescribed across both the previous reviews and this study. For resistance exercise, the previous reviews commonly reported intensities of 30–50% of one-repetition maximum (1RM) or Borg scale ratings of 15–17, whereas this study prescribed light weights (e.g., 1–3 kg) or applied 50% of 1RM, 10RM, 50–100% of 10-repetition maximum (10RM), or Borg scale ratings of 9–17. Similarly, reported intensities for respiratory muscle training in the previous reviews ranged from light loads (1–2 kg) to 60–63% of maximal inspiratory pressure, which were comparable with those applied in this study. The total duration per session ranged from 20 to 90 min in the previous reviews and from 30 to 90 min in this study, with frequencies of 2–5 days/week and 2–6 days/week, respectively. Regarding the setting, center-based programs were most common, followed by telerehabilitation and home-based interventions. Despite these variations, the overall findings remained broadly comparable.

However, a further subgroup analysis showed that longer interventions (>6 weeks) may yield even greater improvements than shorter interventions (<6 weeks) (91.66 *vs*. 71.08 m; Supplementary Figure 1), consistent with the dose–response relationship frequently observed in cardiopulmonary rehabilitation. Mechanistically, enhanced exercise tolerance may stem from improved ventilatory mechanics, increased peripheral oxygen utilization, and reductions in exertional dyspnea (35). Moreover, exercise setting was another factor affecting the 6MWD; the findings indicated that center-based programs resulted in greater improvements in 6MWD than home-based programs (92.5 *vs*. 66.4 m; Supplementary Figure 2), consistent with findings from a recent meta-analysis (35). The greater effectiveness observed in non-home-based programs may be attributable to several factors. Supervised settings allow for closer monitoring, real-time feedback, and individualized adjustment of exercise intensity, which is a key determinant of physiological adaptation. In addition, direct professional guidance may enhance motivation, adherence, and compliance with prescribed training protocols. In contrast, home-based programs often rely on self-management, which may lead to variability in exercise intensity, technique, and overall training dose. A sensitivity analysis was also performed in this study and showed a greater increase in 6MWD after excluding the study with the youngest patients (13). The findings contrast with previous evidence reporting greater improvements in younger adults, attributed to their higher exercise adherence compared with older individuals (35). In this sample, which consisted predominantly of older adults, the larger improvement observed may reflect their greater baseline impairments, allowing exercise interventions to produce more pronounced functional gains than in younger adults who typically begin with higher physical capacity.

Pulmonary function in this study was assessed using FVC and FEV_1_. The findings were consistent with a previous study in patients with COPD (33), which reported an improvement in FEV_1_ (WMD = 0.20 L), comparable with the change observed in this analysis (MD = 0.13 L). Meanwhile, a previous study in individuals with COVID-19 (7) reported no significant change in FVC (MD = 0.12 L), which contrasts with the findings showing a statistically significant improvement (MD = 0.11 L). This discrepancy may be attributed to differences in study populations, as the previous study included both acute and chronic COVID-19 cases, whereas this investigation focused exclusively on patients with post-acute COVID-19 condition.

The MCID for FEV_1_ in individuals with respiratory disease has been reported as approximately 100 mL (36). Therefore, the improvement observed in this study may represent a clinically meaningful enhancement in lung function, even though the magnitude of change was smaller than that reported in many COPD rehabilitation trials. This attenuated response is unsurprising, given that a substantial proportion of participants in this study were older adults with multiple comorbidities. Nevertheless, modest improvements in lung volumes may reflect recovery from post-infectious inflammation, enhanced respiratory muscle conditioning, increased aerobic capacity, and increased thoracic mobility promoted by breathing exercises, inspiratory muscle training (IMT), and aerobic exercise (37, 38). The lack of improvement in FEV_1_/FVC is consistent with the hypothesis that patients with post-COVID-19 condition may produce a mixed pattern of ventilatory impairment, with restrictive changes predominating over airflow obstruction in many patients, as reported in previous systematic reviews (39). Together with improvements in exercise capacity (6MWD) and pulmonary function (e.g., FEV_1_ and FVC), these changes may contribute to reductions in dyspnea (mMRC score), physical pain, and overall enhancements in physical and mental health as well as emotional wellbeing. Although the study by Li et al. (28), which used the SF-12 (physical component summary and mental component summary), was not pooled due to scoring differences, it demonstrated higher physical and mental component scores in the intervention group, indicating a consistent beneficial effect. These findings align with previous studies reporting similar improvements across these symptom domains (7, 35).

Although this meta-analysis demonstrated consistent improvements in exercise capacity, pulmonary function, and symptoms across studies, it remains difficult to determine whether the type of exercise program influenced the magnitude of benefit. This is because most trials employed broadly similar interventions, with nine incorporating aerobic training, nine including resistance exercises, and several including breathing exercises (e.g., IMT, diaphragmatic breathing, and thoracic mobility/expansion exercises), often delivered in combination. Thus, the similarity in treatment effects across studies may reflect the predominance of aerobic- and resistance-based programs rather than true equivalence between different exercise modalities. The findings are consistent with previous systematic reviews reporting that aerobic, resistance, and IMT represent the most effective exercise components for post-COVID-19 rehabilitation (37). In addition, variations in exercise-intensity protocols across studies warrant caution when interpreting the pooled results, as intensity is a key determinant of physiological adaptation. Although four studies explicitly reported that no adverse events were observed, safety data were not reported in the remaining studies. The absence of reporting does not necessarily indicate the absence of adverse events, highlighting a gap in safety evidence. Therefore, firm conclusions regarding the overall safety of exercise-based rehabilitation in this population cannot be drawn. This concern may be particularly relevant for home-based programs, where supervision is limited, although ensuring adequate exercise adherence in such settings remains challenging. Taken together, these considerations suggest that exercise dose and adherence may represent more critical determinants of effectiveness than the specific exercise modality selected.

### 4.1 Strengths and limitations

A major strength of this meta-analysis is the focus on interventions lasting 6 weeks or longer. This threshold aligns with well-established physiological principles describing the timeline required for meaningful cardiorespiratory adaptations, particularly improvements in aerobic metabolism, ventilatory efficiency, and peripheral muscle function. In addition, the methodological quality of the included studies—restricted to randomized controlled trials—was high, with most trials scoring within the good-to-excellent range on the PEDro scale. However, several limitations must be acknowledged. First, the search was restricted to three major databases and to English-language publications, which may have introduced language bias and resulted in the exclusion of relevant studies published in other languages. Second, despite the focus on interventions of at least 6 weeks, notable heterogeneity persisted in exercise type, intensity, exercise settings, and patient characteristics. Third, several outcomes were reported in only two to three studies, reducing statistical precision. Furthermore, individuals with post-COVID-19 represent a clinically heterogeneous population with fluctuating and multisystem symptoms. Individual responsiveness to exercise may therefore vary according to comorbidities, initial disease severity, and persistent systemic inflammation. Fourth, publication-bias assessment was limited due to fewer than 10 included studies per outcome. Consequently, funnel plots and Egger’s test had low statistical power, and publication bias cannot be ruled out. Finally, to enhance internal validity, trials involving specific comorbid populations or structured co-interventions were excluded. However, in real-world clinical practice, many patients with post-COVID-19 condition present with multimorbidity and receive concurrent treatments as part of multidisciplinary care. Therefore, caution is warranted when extrapolating these findings to broader and more complex patient populations.

### 4.2 Future directions

Further research should aim to standardize exercise prescriptions, identify optimal training intensities for different post-COVID-19 condition phenotypes, and determine which subgroups benefit the most (e.g., based on disease severity, age, or symptom clusters). Long-term follow-up is also needed to evaluate the sustainability of exercise-induced improvements. In addition, although center-based exercise generally produced greater improvements in some outcomes, telerehabilitation demonstrated promising results in several included trials and therefore warrants further exploration as an accessible rehabilitation modality.

## 5 Conclusion

Exercise interventions lasting at least 6 weeks appear to be effective in improving exercise capacity and pulmonary function, mitigating dyspnea and pain, and enhancing broader physical and health-related outcomes among individuals with post-COVID-19 condition.

## Data Availability

The original contributions presented in the study are included in the article/Supplementary material, further inquiries can be directed to the corresponding author.

## Supplementary material

SUPPLEMENTARY FIGURE 1 Forest plot of the subgroup analysis (6 weeks *vs*. > 6 weeks) of the seven studies reporting 6MWD (m) comparing exercise and control groups.

SUPPLEMENTARY FIGURE 2 Forest plot of the subgroup analysis (home-based *vs*. non-home-based) of the seven studies reporting 6MWD (m) comparing exercise and control groups.

SUPPLEMENTARY FIGURE 3 Forest plot from the sensitivity analysis of 6MWD, excluding the study by Alsharidah et al. (2023) (13), which included the youngest population, comparing the exercise and control groups.

SUPPLEMENTARY FIGURE 4 Funnel plot of the seven studies reporting 6MWD.

SUPPLEMENTARY FIGURE 5 Funnel plot of the seven studies reporting FVC.

SUPPLEMENTARY FIGURE 6 Funnel plot of the six studies reporting FEV_1_.

SUPPLEMENTARY FIGURE 7 Funnel plot of the five studies reporting mMRC.
