# Supplementary figures and images for: Effects of exercise programs on cardiopulmonary function and signs and symptoms in patients with post-COVID-19 condition: a systematic review and meta-analysis
Source: Front Med (Lausanne). 2026 Mar 17;13:1772741. doi: 10.3389/fmed.2026.1772741 (PMC13035521; doi:10.3389/fmed.2026.1772741)

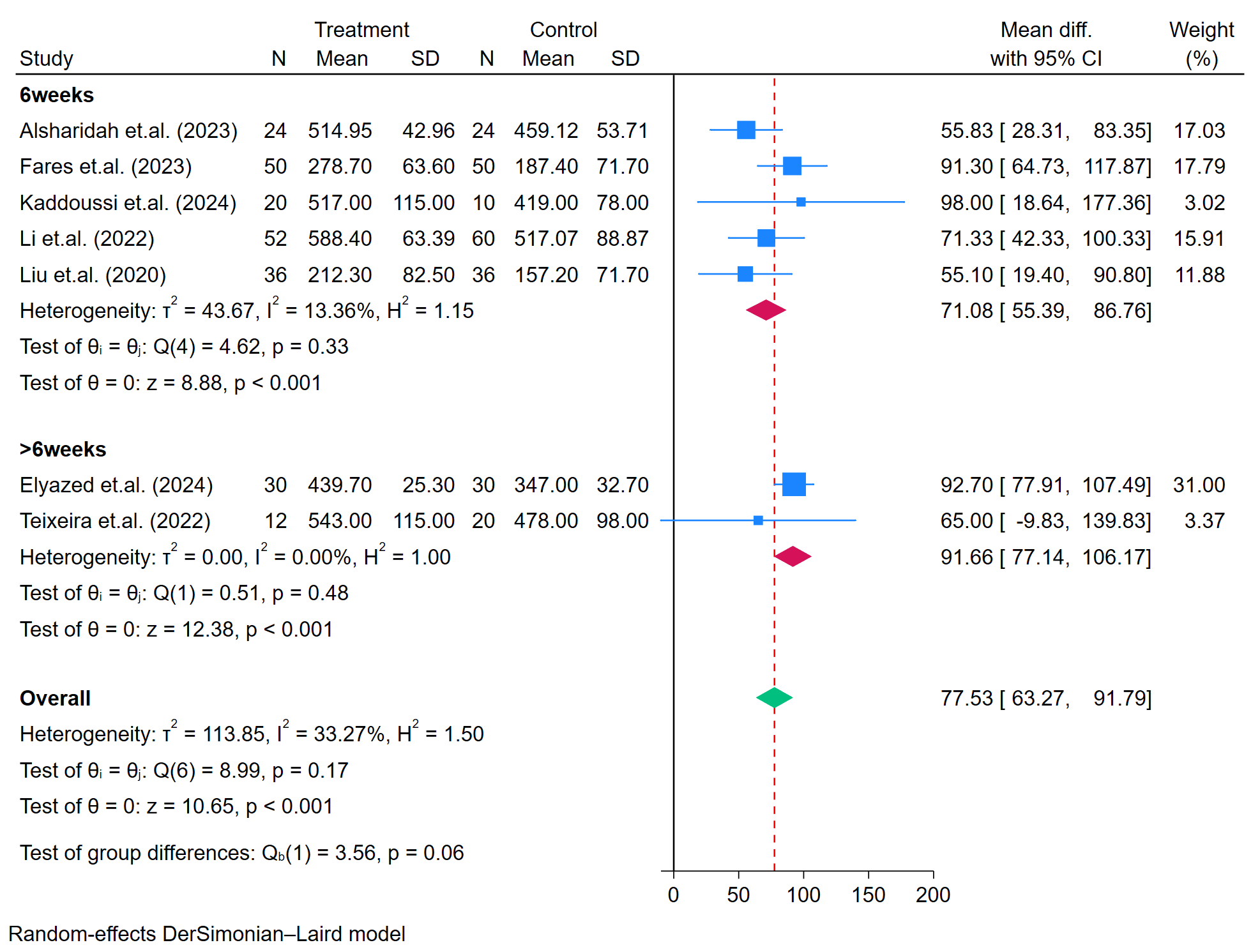

Supplement: SUPPLEMENTARY FIGURE 1 — Forest plot of the subgroup analysis (6 weeks vs. > 6 weeks) of the seven studies reporting 6MWD (m) comparing exercise and control groups. [file Image_1.tif]

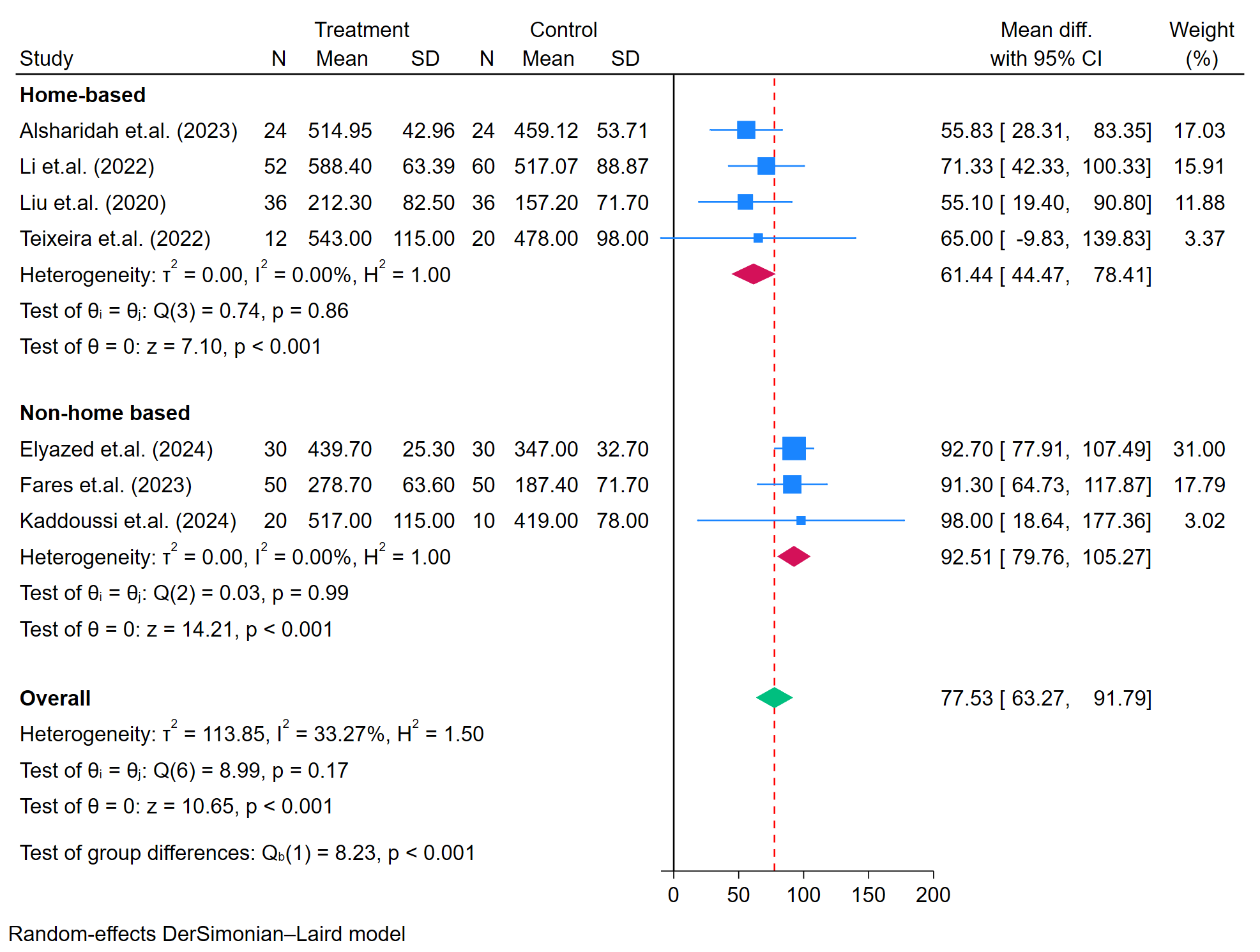

Supplement: SUPPLEMENTARY FIGURE 2 — Forest plot of the subgroup analysis (home-based vs. non-home-based) of the seven studies reporting 6MWD (m) comparing exercise and control groups. [file Image_2.tif]

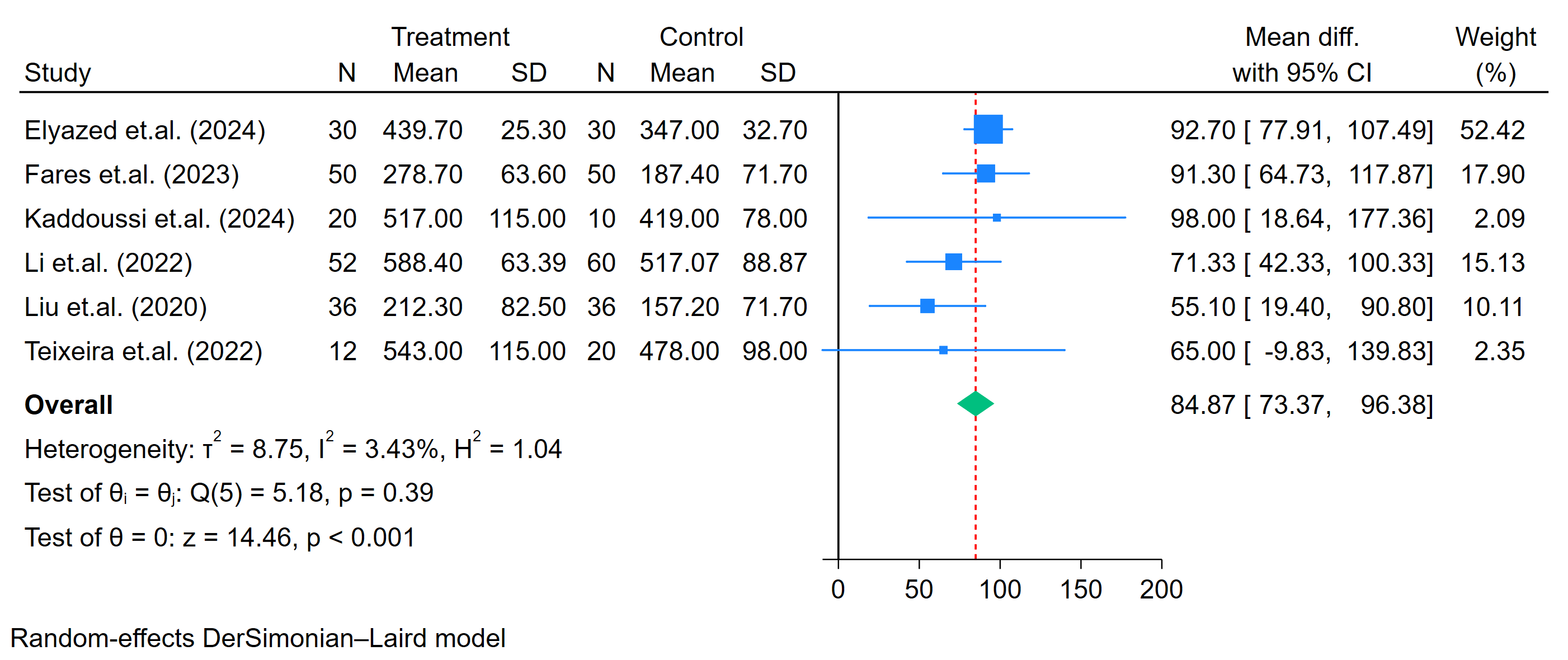

Supplement: SUPPLEMENTARY FIGURE 3 — Forest plot from the sensitivity analysis of 6MWD, excluding the study by Alsharidah et al. (2023) (13), which included the youngest population, comparing the exercise and control groups. [file Image_3.tif]

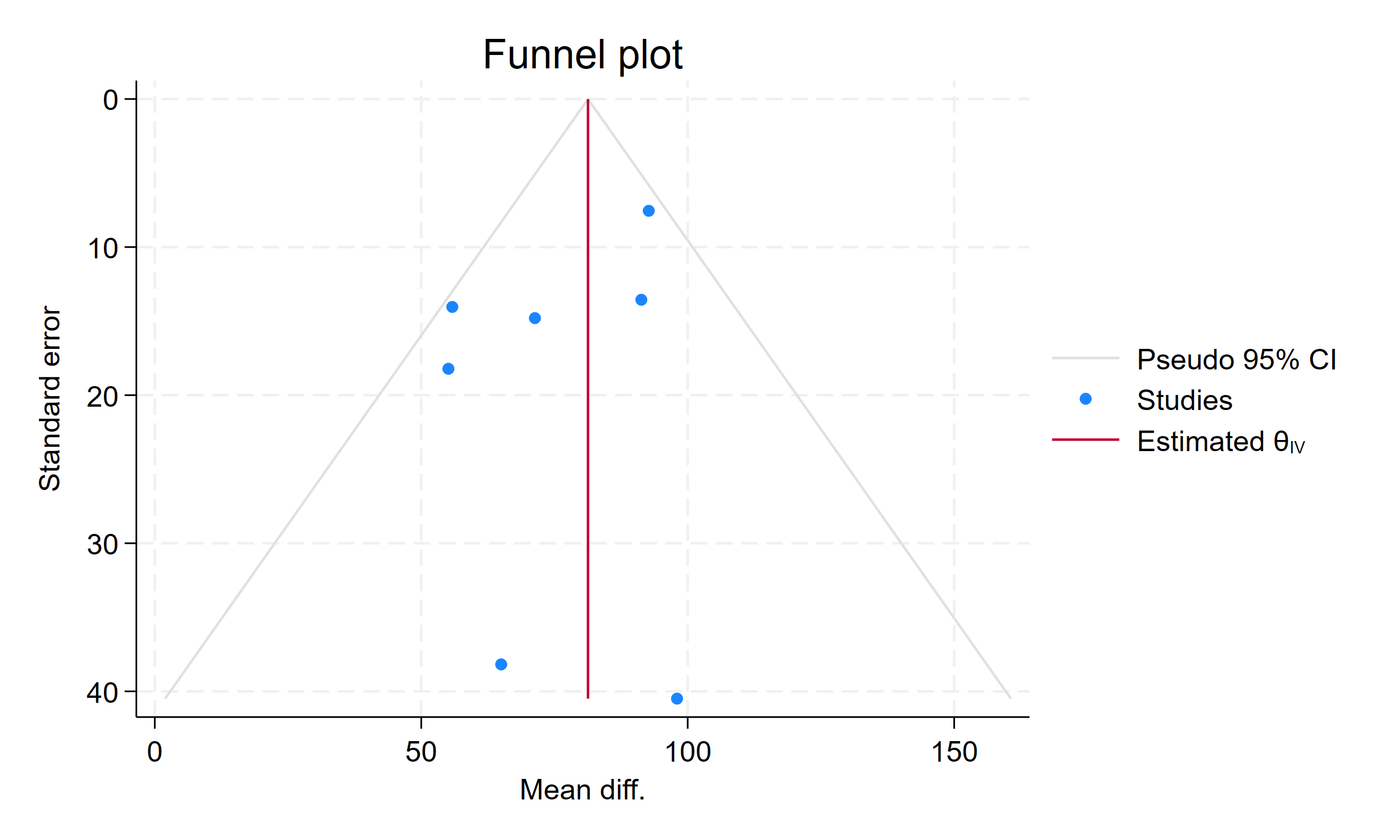

Supplement: SUPPLEMENTARY FIGURE 4 — Funnel plot of the seven studies reporting 6MWD. [file Image_4.tif]

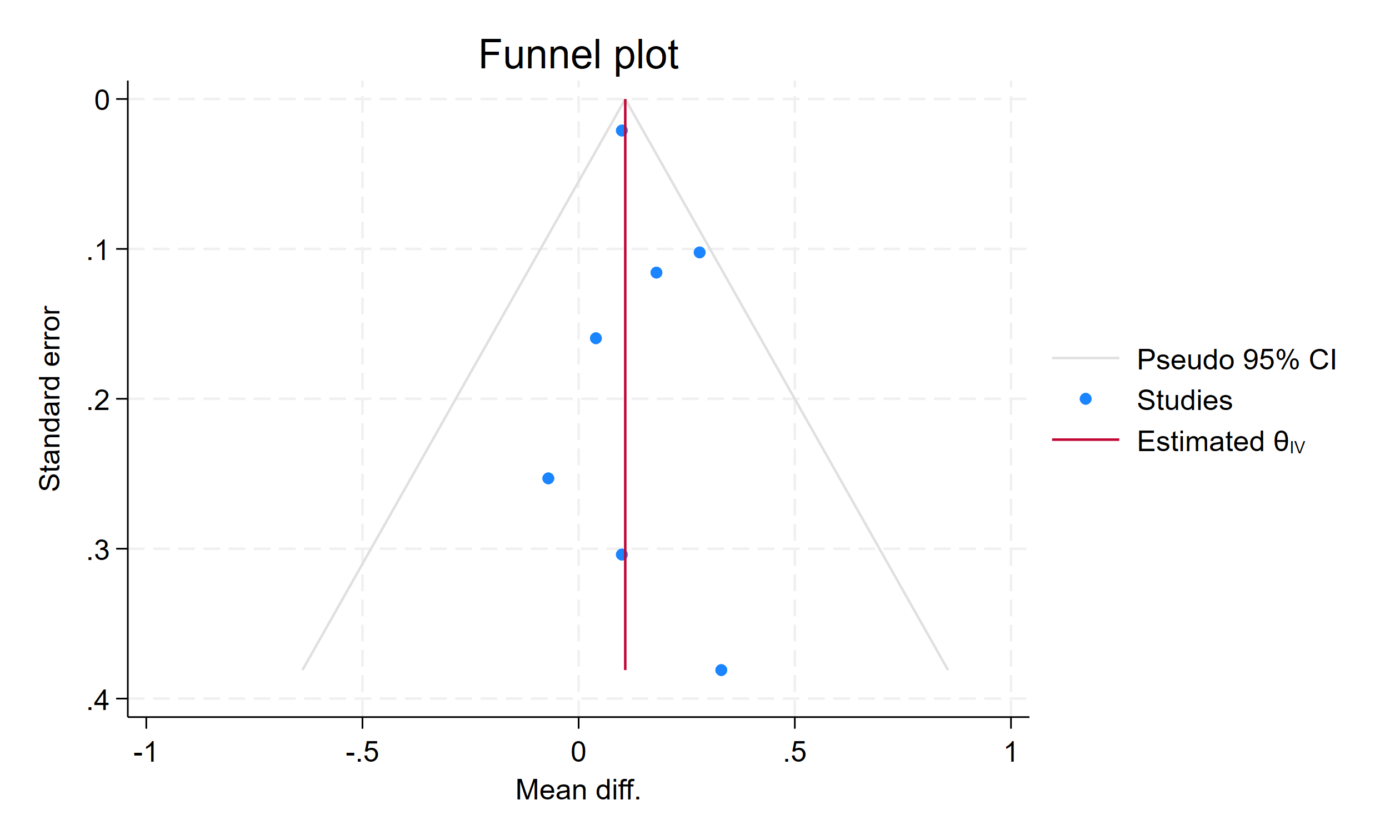

Supplement: SUPPLEMENTARY FIGURE 5 — Funnel plot of the seven studies reporting FVC. [file Image_5.tif]

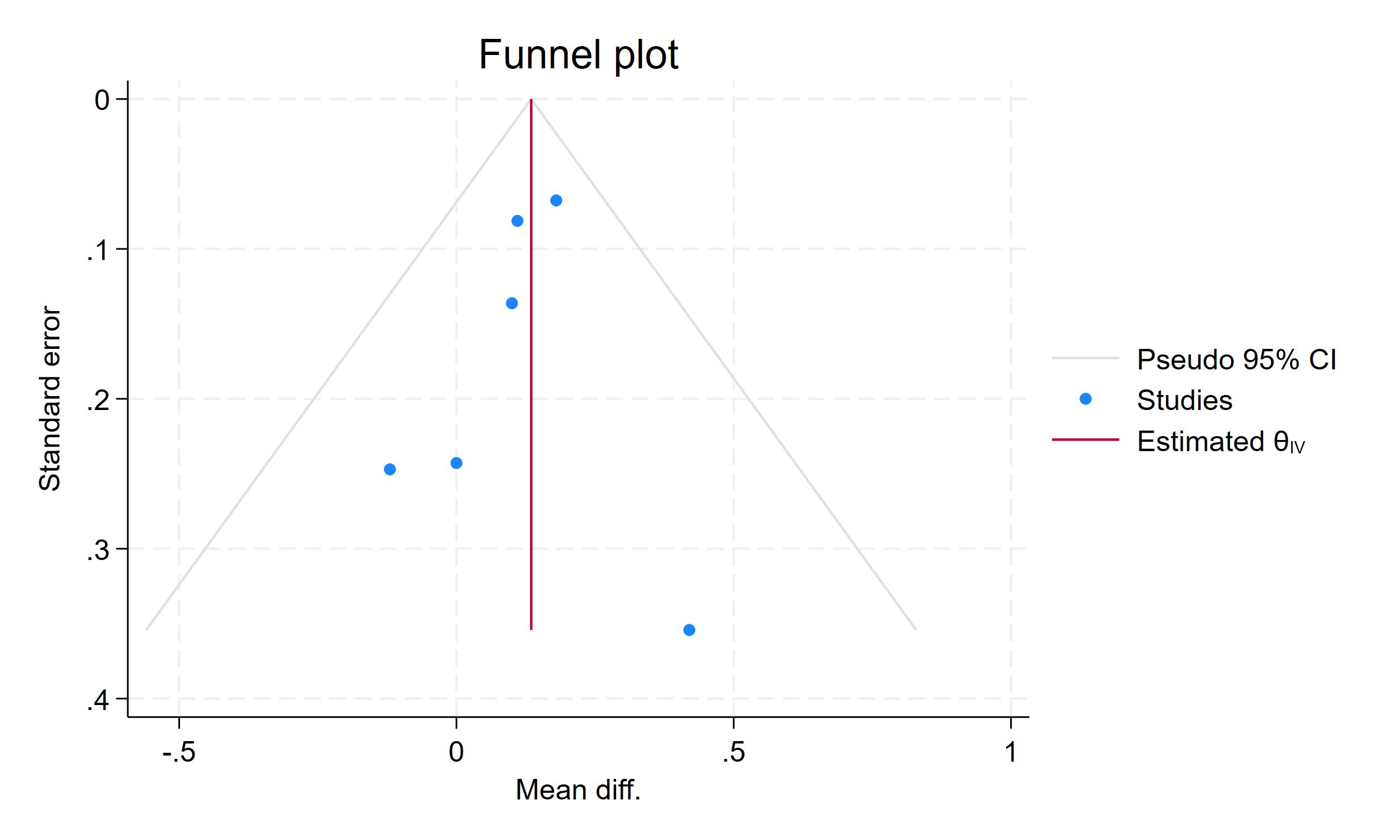

Supplement: SUPPLEMENTARY FIGURE 6 — Funnel plot of the six studies reporting FEV1. [file Image_6.tif]

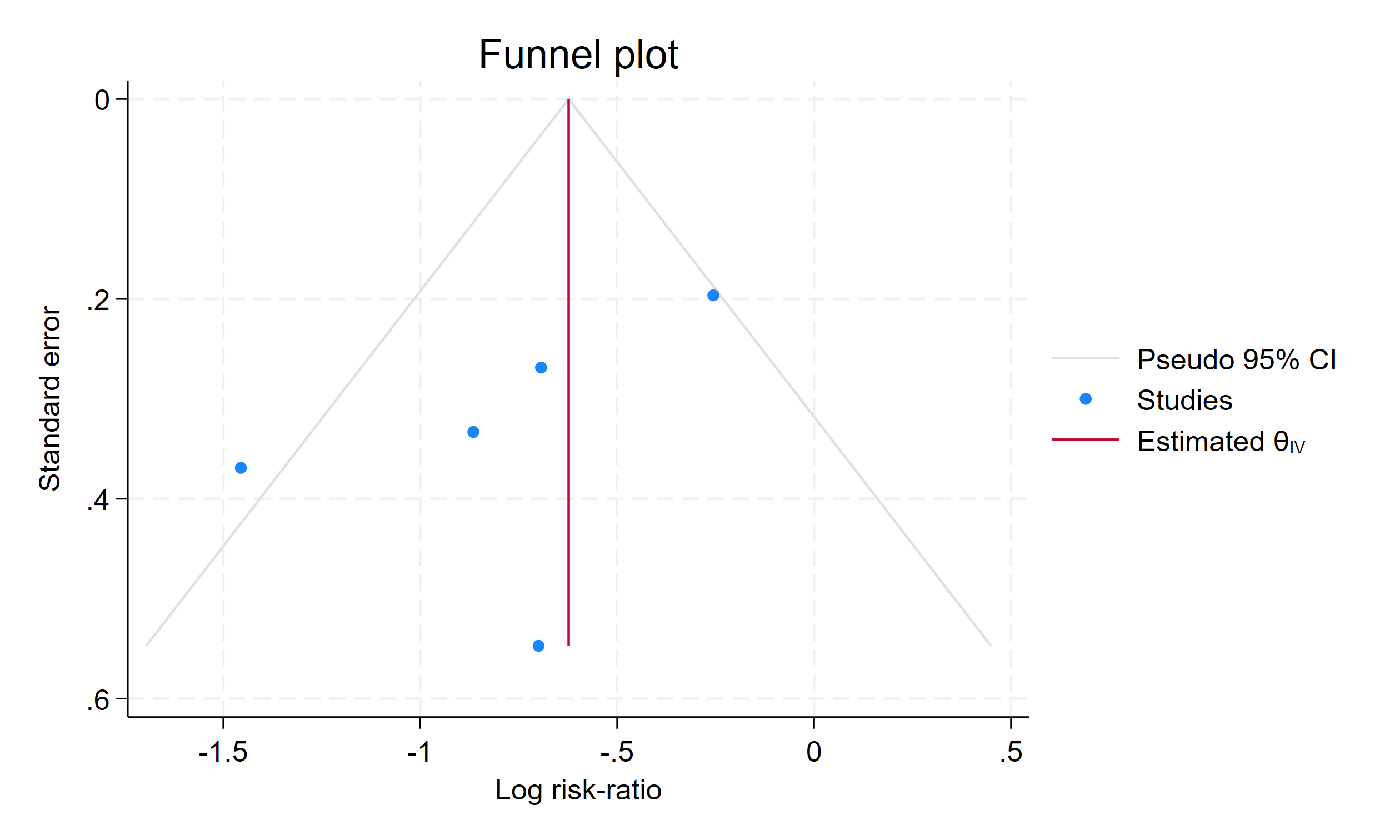

Supplement: SUPPLEMENTARY FIGURE 7 — Funnel plot of the five studies reporting mMRC. [file Image_7.tif]
