# Supplementary material for: Effects of exercise programs on cardiopulmonary function and signs and symptoms in patients with post-COVID-19 condition: a systematic review and meta-analysis
Source: Front Med (Lausanne). 2026 Mar 17;13:1772741. doi: 10.3389/fmed.2026.1772741 (PMC13035521; doi:10.3389/fmed.2026.1772741)
Supplement: Supplementary file 8 [file Table_1.docx]

**Supplementary Table 1** Full electronic search strategies for all databases.

All searches were conducted on April 22, 2024. Searches were limited to English-language publications and human studies. No date restrictions were applied.

| **Keywords** | **Database** | **Number of papers** |
| --- | --- | --- |
| ((((("Controlled Clinical Trial" [Publication Type]) OR "Clinical Trial" [Publication Type]) OR "Randomized Controlled Trial" [Publication Type]) AND (("Respiratory Function Tests"[Mesh]) OR "Signs and Symptoms"[Mesh])) AND (("Exercise"[Mesh]) OR ( "Telerehabilitation"[Mesh] OR "Rehabilitation"[Mesh] ))) AND (("COVID-19"[Mesh] OR "SARS-CoV-2"[Mesh]) OR "Coronavirus"[Mesh]) | Pubmed | 65 |
| ( ( TITLE-ABS-KEY ( covid-19 ) OR TITLE-ABS-KEY ( sars-cov-2 ) OR TITLE-ABS-KEY ( coronavirus ) ) ) AND ( ( TITLE-ABS-KEY ( respiratory AND function AND test ) OR TITLE-ABS-KEY ( signs AND symptoms ) ) ) AND ( ( TITLE-ABS-KEY ( exercise ) OR TITLE-ABS-KEY ( rehabilitation ) OR TITLE-ABS-KEY ( telerehabilitation ) ) ) AND ( ( TITLE-ABS-KEY ( controlled AND clinical AND trial ) OR TITLE-ABS-KEY ( randomized AND controlled AND trial ) OR TITLE-ABS-KEY ( clinical AND trial ) ) ) | Scopus | 117 |
| covid 19.mp. or coronavirus disease 2019 or coronavirus.mp. or Coronavirinae or coronavirus disease 2019/ or SARS-COV-2.mp. AND Exercise.mp. or exercise or physical exercise.mp. or exercise or rehabilitation.mp. or pulmonary rehabilitation or rehabilitation or rehabilitation program.mp. or rehabilitation AND pulmonary function.mp. or lung function or lung function.mp. or lung function or (signs and symptoms).mp. [mp=title, abstract, heading word, drug trade name, original title, device manufacturer, drug manufacturer, device trade name, keyword heading word, floating subheading word, candidate term word] AND randomized controlled trial/ or clinical trial/ or randomi* control* trial.mp. or clinical trial.mp. or clinical trial/ or clinical trial/ or randomized controlled trial/ or RCT.mp. | Embase | 133 |
| SARS-CoV-2/ or Coronavirus Infections/ or COVID-19/ or covid 10.mp. or coronavirus.mp. or Coronavirus or SARS-CoV-2.mp. or SARS-CoV-2 AND Exercise Therapy/ or Exercise/ or exercise.mp. or physical exercise.mp. or Exercise or Rehabilitation/ or rehabilitation.mp. or Exercise Therapy/ or rehabilitation program.mp. AND pulmonary function.mp. or Respiratory Function Tests/ or lung function.mp. or Respiratory Function Tests or (signs and symptoms).mp. [mp=title, book title, abstract, original title, name of substance word, subject heading word, floating sub-heading word, keyword heading word, organism supplementary concept word, protocol supplementary concept word, rare disease supplementary concept word, unique identifier, synonyms, population supplementary concept word, anatomy supplementary concept word] AND Randomized Controlled Trials as Topic/ or randomize control trial.mp. or clinical trial.mp. or Clinical Trial/ or Randomized Controlled Trials as Topic/ or RCT.mp. | Medline (via Ovid) | 34 |
| **Total** |  | 349 |
