# Supplementary material for: Effects of exercise programs on cardiopulmonary function and signs and symptoms in patients with post-COVID-19 condition: a systematic review and meta-analysis
Source: Front Med (Lausanne). 2026 Mar 17;13:1772741. doi: 10.3389/fmed.2026.1772741 (PMC13035521; doi:10.3389/fmed.2026.1772741)
Supplement: Supplementary file 10 [file Table_3.docx]

**Supplementary Table 3** GRADE assessment of the certainty of evidence for each outcome.

| **Outcomes** | **Quality assessment** | | | | | | **Summary of findings** | |
| --- | --- | --- | --- | --- | --- | --- | --- | --- |
| No. of the studies (no.of participants) | Study design (all RCTs) | Study limitations (Pedro score, rated) | Consistency (I^2^) | Directness (PICO clarity) | Precision (95%CI), n | Publication bias | Effect size (MD or RR) (95% CI) | Certainty |
| 6MWT | | | | | | | | |
| 7 (454) | High | Good | Yes (I^2^=33%) | Yes | Yes | Undetected | 77.53 m. (63.27, 91.79) | ⊕⊕⊕⊕ = High |
| FVC | | | | | | | | |
| 7 (443) | High | Good | Yes (I^2^=0%) | Yes | Yes | Undetected | 0.11 L (0.07, 0.15) | ⊕⊕⊕⊕ = High |
| FEV_1_ | | | | | | | | |
| 6(343) | High | Good | Yes (I^2^=0%) | Yes | Yes | Undetected | 0.13 L (0.04, 0.23) | ⊕⊕⊕⊕ = High |
| mMRC | | | | | | | | |
| 5(357) | High | Good | No (I^2^=57%) | Yes | Yes | Undetected | RR 0.48* (0.31, 0.73) | ⊕⊕〇〇 = Moderate |
| Physical pain | | | | | | | | |
| 2(120) | High | Good | Yes (I^2^=2%) | Yes | No | Do not assess | 16.93 point (14.85, 19.01) | ⊕⊕〇〇 = Moderate |
| Physical health | | | | | | | | |
| 3(187) | High | Good | No (I^2^=95%) | Yes | No | Do not assess | 7.24 points (3.20, 11.27) | ⊕⊕〇〇 = Moderate |
| Mental health | | | | | | | | |
| 2(120) | High | Good | Yes (I^2^=0%) | Yes | No | Do not assess | 9.01 points (2.09, 15.93) | ⊕⊕〇〇 = Moderate |
| Emotional role | | | | | | | | |
| 2(120) | High | Good | Yes (I2=0%) | Yes | No | Do not assess | 15.19 points (12.88, 17.51) | ⊕⊕〇〇 = Moderate |

* = risk ratio
